# Mammals in Tawny Owl (*Strixaluco*) pellets from Kharkiv Region, Ukraine

**DOI:** 10.3897/BDJ.11.e98772

**Published:** 2023-01-20

**Authors:** Yehor Yatsiuk, Nataliia Brusentsova, Yuliya Filatova

**Affiliations:** 1 Institute of Ecology and Earth Sciences, University of Tartu, Tartu, Estonia Institute of Ecology and Earth Sciences, University of Tartu Tartu Estonia; 2 Tuzlivski Lymany National Nature Park, Tatarbunary, Odesa region, Ukraine Tuzlivski Lymany National Nature Park Tatarbunary, Odesa region Ukraine; 3 Slobozhanskyi National Nature Park, Krasnokutsk, Kharkiv Region, Ukraine Slobozhanskyi National Nature Park Krasnokutsk, Kharkiv Region Ukraine; 4 Independent researcher, Kharkiv, Ukraine Independent researcher Kharkiv Ukraine

**Keywords:** owl pellets, diet, mammals, rodents, Gliridae, Muridae, Cricetidae, Leporidae, Mustelidae, Vespertilionidae, Erinaceidae, Talpidae, Soricidae, Tawny owl, *Strixaluco*

## Abstract

**Background:**

The Tawny owl (*Strixaluco*) is a common owl species in Europe, demonstrating generalist diet strategy. Its main prey are small rodents and numerous studies show that the composition of its diet broadly reflects changes in prey species abundance in its habitats. Due to strictly sedentary habits of adult birds and their year-round territoriality, it is possible to locate habitats of their prey with a precision of several hundred metres. Analysis of owl pellets is a traditional method in faunistic studies to provide data on distribution of small mammals, especially cryptic species which are hard to be found using trapping.

**New information:**

Here, we present a dataset on mammals found in Tawny owl pellets collected during up to 13-year studies in the Kharkiv Region, Ukraine in three territories. Data from two territories were collected in a systematic way and allowed us to make analysis of seasonal, year-to-year and habitat variability in the Tawny owl diet and local mammal species composition.

## Introduction

The Tawny owl (*Strixaluco*) is a species with generalist diet habits, widespread in Europe. Its main prey are small rodents, but also includes a number of other mammal groups, birds, reptiles, amphibians and invertebrate species. It has the most diverse studied diet across all European owl species (Galeotti 2001). Birds are highly territorial throughout the year (Southern 1970) and have a set of preferred shelters, predominantly in tree cavities (Yatsiuk and Wesołowski 2021), within their individual territories, under which they leave pellets. It had been shown that Tawny owl pellet content broadly reflects local fauna of small mammals and follows fluctuations of small rodents, but owls readily switch to alternative prey (insectivores, small birds, amphibians) when rodent numbers decrease (Southern 1954, Jędrzejewski et al. 1994, Jędrzejewski et al. 1996, Galeotti 2001).

Analysis of pellets is a traditional method for analysing the owl diet, but also it provides additional data on the distribution of small mammals, especially cryptic species which are hard to find using trapping (Cserkész et al. 2009, McDonald et al. 2013, Biedma et al. 2019).

In the eastern part of Ukraine, on the border between East European forest-steppe and Pontic steppe ecoregions (Olson et al. 2001), the Tawny owl is the only forest owl species. Here, it inhabits all types of woodlands and reaches highest densities in Oak (*Quercusrobur*)-dominated broadleaved forests (Yatsiuk 2020). Most published works analysing owl pellet content in the region provide results of short trials and are based on small samples.

Here, we present the data collected in three areas in Kharkiv Region (Ukraine): in the National Nature Park (NNP) Homilsha Forest (broadleaved forest, 2005-2014) and suburban Kharkiv Forest Park (broadleaved forest, 2011-2015) with additional data collected in the National Nature Park (NNP) Slobozhanskyi (pine and broadleaved forests, 2013-2017). In the first two territories, sampling was made as part of a monitoring programme including annual bird censuses, control of nest boxes and all known tree cavities. Pellets were found under nest boxes or tree cavities or inside them. All pellets were collected during each check which allowed us to separate samples by seasons. Mapping of territorial pairs gave the density of 2.3 pairs/km^2^ in broadleaved forests which means that prey items found in pellets may have been collected within 350-400 m radius around each sampling point, which allows habitat analysis. Thus, the dataset allows regional, habitat, annual and seasonal analysis of pellet contents.

A part of the data included in the current dataset (content of 1648 pellets collected between 2007 and 2012 in the NNP Homilsha Forest) had been used in a previous publication (Yatsiuk and Filatova 2017).

## Project description

### Title

Monitoring of Tawny owl (*Strixaluco*) in Kharkiv Region, Ukraine

### Personnel

Yehor Yatsiuk, Nataliia Brusentsova, Yuliya Filatova

## Sampling methods

### Sampling description

Field surveys were made between April 2004 and November 2014 in NNP Homilsha Forest, between May 2011 and March 2015 in Kharkiv Forest Park and between April 2013 and May 2017 in NNP Slobozhanskyi.

In NNP Homilsha Forest and in Kharkiv Forest Park, pellets were collected during regular checks of nest boxes and all known tree cavities. In the first area, all sites were visited three times a year: in mid-April, beginning of July and late November or early December. In the second area, all sites were visited two times a year: in April-May and in November-December. During each check, we collected all pellets from each site which allowed us to separate samples by seasons. In NNP Slobozhanskyi, pellets were sampled only occasionally under cavity trees or on the ground.

Collected pellets were macerated in water, then the wool/fur of prey was removed and the bones were collected and dried (Novikov 1949). Samples of pellets from each locality collected at one time were processed together, with number of prey specimens determined for each sample (Yatsiuk and Filatova 2017).

Prey species were identified following taxonomic keys (Nebogatkin 1987, März 2007, Voronetsky and Kuzmenko 2013). The maximal number of individuals for each vertebrate prey species was determined as the maximal number of upper and lower jaws.

When it was impossible to identify prey remains to species level, they were assigned to higher-level genus or family level. Remains of voles from *Microtus* “*arvalis*” group were treated as the East European vole (*Microtuslevis* Miller, 1908) in accordance with earlier karyological studies in the studied region (Zagorodniuk 1993, Zagorodniuk 2008). Unidentified remains of mice (assigned to category Muridae) belonged to each of two genera, *Mus* or small *Apodemus*. Category *Apodemus* includes either *A.sylvaticus* or *A.uralensis* as both species can occur in the region (Naglov 1996, Zagorodniuk 2020), but it was not possible to distinguish between them, based on bone remains present. Two remains of *Mustela* sp. probably belong to *M.nivalis*. Remains of Soricidae represent small shrews, either *Sorex* or *Crocidura*.

### Quality control

Pellet samples were identified by the authors with consultations from the specialists from the Crimea Plague Control Station, Kharkiv Sanitary-Epidemiological Station and Kharkiv National University.

## Geographic coverage

### Description

**Figure 1.**
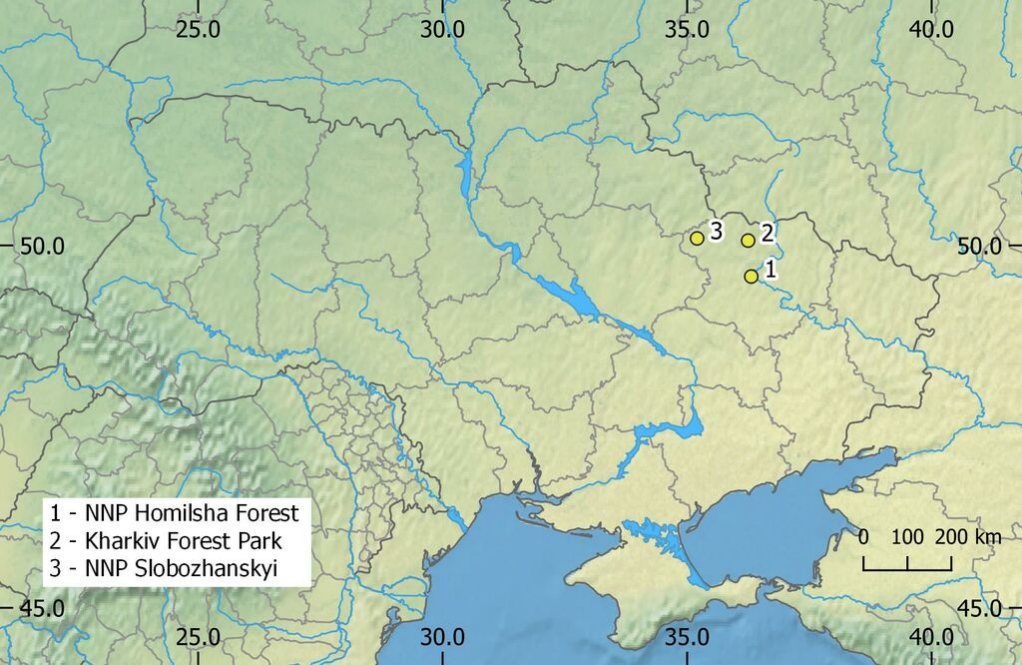
Location of study sites.

The study area is located in Kharkiv Region, Ukraine, at the southern border of the East European forest-steppe ecoregion (Fig. 1) (Olson et al. 2001). The climate of the region is moderately-continental with mean air temperatures between +21°C in July and -7°C in January and average annual precipitation 540 mm (Golikov et al. 2011). During the study period, the average duration of snow cover was 90-100 days, with more stable cover on elevated sites and frequent thawing in river valleys. The terrain of the region is undulating plains with elevations between 90 and 220 m. The total forest cover is 13.16% in the northern forest-steppe part of the region and includes two main forest types. Broadleaved forests grow on rich clay soils on elevated sites and cover 9.7% of the total area. The main tree species is oak (*Quercusrobur*) with a high proportion of lime (*Tiliacordata*), maples (*Acerplatanoides* and *A.campestre*) and ash (*Fraxinusexcelsior*). Coniferous forests grow on sandy river terraces and cover 3.4% of the total area. The dominating tree species is pine (*Pinussylvestris*) with a small share of deciduous stands (mainly aspen *Populustremula* and black alder *Alnusglutionsa* in carrs); broadleaved species occur in a relatively small proportion.

The first study area in NNP Homilsha Forest is located in the broadleaved forest along Siversky Donets River. Clearcut-based silviculture with 90-120 years rotations was used here up to 2007 when it ceased after the creation of the National Park. Currently, most of forest stands here are of natural origin and old-growth forest with the age of 100-150 years covering about half of its territory. The study area is surrounded by rural areas and riverine habitats.

The second study area in Kharkiv Forest Park is located at the northern part of the city. It is a part of a larger forest continuing for about 20 km to the north from Kharkiv. No clearcuts are done in this forest; however, thinnings and sanitary cuttings are practised. The mean stand age is 60–100 years. There is a gradient of transformation from recreational grounds in the southern part to semi-natural stands in the northern part. The study area is mostly surrounded by built-up areas.

The third study area is located in pine and broadleaved forests along the Merla River valley in NNP Slobozhanskyi. Pine forests here include frequent wet depressions and fens. Before the creation of the National Park in 2009, clearcut-based silviculture with 90-110 years rotations was used here. Currently, most of pine stands here are of planted origin with mean age 50-90 years. The mean age of broadleaved forest is 80-120 years and clearcut-based silviculture has been similarly ceased here since 2009.

### Coordinates

49.554 and 50.127 Latitude; 35.160 and 36.361 Longitude.

## Taxonomic coverage

### Description

The presented dataset covers 23 mammal species from six orders and nine families with 1213 occurrences in total.

### Taxa included

**Table:** 

Rank	Scientific Name	
kingdom	Animalia	
phylum	Chordata	
class	Mammalia	
order	Soricomorpha	
family	Soricidae	
species	*Sorexaraneus* Linnaeus, 1758	
species	*Sorexminutus* Linnaeus, 1766	
species	*Crocidurasuaveolens* (Pallas, 1811)	
species	*Neomysfodiens* (Pennant, 1771)	
family	Talpidae	
species	*Talpaeuropaea* Linnaeus, 1758	
order	Chiroptera	
family	Vespertilionidae	
genus	*Myotis* Kaup, 1829	
genus	*Pipistrellus* Kaup, 1829	
species	*Vespertiliomurinus* Linnaeus, 1758	
species	*Nyctalusnoctula* (Schreber, 1774)	
species	*Plecotusauritus* (Linnaeus, 1758)	
order	Carnivora	
family	Mustelidae	
genus	*Mustela* Linnaeus, 1758	
order	Lagomorpha	
family	Leporidae	
species	*Lepuseuropaeus* Pallas, 1778	
order	Rodentia	
family	Gliridae	
species	*Dryomysnitedula* (Pallas, 1778)	
family	Cricetidae	
species	*Cricetulusmigratorius* (Pallas, 1773)	
species	*Myodesglareolus* (Schreber, 1780)	
species	*Arvicolaamphibius* (Linnaeus, 1758)	
genus	*Microtus* Schrank, 1798	
species	*Microtuslevis* Miller, 1908	
species	*Microtussubterraneus* (Selys-Longchamps, 1836)	
species	*Microtusoeconomus* (Pallas, 1776)	
family	Muridae	
genus	*Rattus* Fischer, 1803	
species	*Rattusnorvegicus* (Berkenhout, 1769)	
species	*Musspicilegus* Petényi, 1882	
species	*Musmusculus* Linnaeus, 1758	
genus	*Apodemus* Kaup, 1829	
species	*Apodemusagrarius* (Pallas, 1771)	
species	*Apodemusflavicollis* (Melchior, 1834)	
species	*Micromysminutus* (Pallas, 1771)	

## Temporal coverage

### Notes

NNP Homilsha Forest: from 05-04-2005 to 22-11-2014

Kharkiv Forest Park: from 13-05-201 to 18-03-2015

NNP Slobozhanskyi: from 19-04-2013 to 01-05-2017

## Usage licence

### Usage licence

Other

### IP rights notes

This work is licensed under a Creative Commons Attribution (CC-BY) 4.0 License.

## Data resources

### Data package title

Mammals in Tawny owl (*Strixaluco*) pellets from Kharkiv Region, Ukraine

### Resource link

https://www.gbif.org/dataset/ce7299c8-a8b9-4d92-9055-cd316b2bf1d2

### Alternative identifiers

https://doi.org/10.15468/9k4wrz

### Number of data sets

1

### Data set 1.

#### Data set name

Mammals in Tawny owl (*Strixaluco*) pellets from Kharkiv Region, Ukraine

#### Data format

Darwin Core; tab separated text file

#### Character set

UTF-8

#### Download URL

https://www.gbif.org/dataset/ce7299c8-a8b9-4d92-9055-cd316b2bf1d2

#### Description

The dataset contains data of mammals found in Tawny owl pellets collected during up to 13-year studies (2005-2017) in Kharkiv Region, Ukraine, in three territories (Yatsiuk et al. 2022). Data from broadleaved forests in National Nature Park Homilsha Forest and Kharkiv Forest Park were collected in a systematic way when all found pellets were removed from each site two or three times a year. Data from broadleaved and pine forests in National Nature Park Slobozhanskyi were sampled one time from each site. Presented data allowed us to make analysis of seasonal, year-to-year and habitat variability in the Tawny owl diet and local mammal species composition.

**Data set 1.** 

Column label	Column description
eventID	Unique identifier for each sampling event: one-time collection of pellets from one point.
samplingProtocol	Description of method used: in all cases, the basis was analysis of owl pellets.
samplingEffort	Additional description of sampling in the context of time and coverage.
sampleSizeValue	Number of pellets collected from each site.
sampleSizeUnit	Units for sampleSizeValue column.
eventDate	Sample collection date.
fieldNotes	Additional description of site where pellets were collected.
country	Country name.
countryCode	Country code.
stateProvince	Name of a region within the country.
locality	Name of a study area: NNP Homilsha Forest, Kharkiv Forest Park, NNP Slobozhanskyi.
locationID	Unique codes for each location: nest boxes, tree cavities or other sites.
decimalLatitude	Decimal coordinates.
decimalLongitude	Decimal coordinates.
geodeticDatum	The geodetic datum for the given decimalLatitude and decimalLongitude.
coordinateUncertaintyInMetres	The horizontal distance in metres from the given decimalLatitude and decimalLongitude describing the smallest circle containing the whole of the Location.
georeferencedBy	Name of person making the georeference.
type	Type of the resource for which information in 'Event' table is given.
occurrenceID	Unique identifier for each species occurrence found in the pellet sample. Based on eventID with added sequential numbers.
basisOfRecord	Recommended best practice is to use the standard label of one of the Darwin Core classes.
organismQuantity	Number of individuals of each species found in each sample
organismQuantityType	Unit used in the 'organismQuantity' field
occurrenceStatus	Status of species occurrence data. Only presence data are given in the dataset.
scientificName	Full scientific name of prey species.
kingdom	Kingdom name.
phylum	Phylum name.
class	Class name.
order	Order name.
family	Family name.
genus	Genus name.
specificEpithet	Specific epithet.
taxonRank	Taxon rank.
recordedBy	Names of persons who collected and identified samples.
